# Syntaxin-6 restricts SARS-CoV-2 infection by facilitating virus trafficking to autophagosomes

**DOI:** 10.1128/jvi.00002-25

**Published:** 2025-04-25

**Authors:** Hao Sun, Qi Yang, Yecheng Zhang, Saisai Cui, Zhe Zhou, Peilu Zhang, Lijia Jia, Mingxia Zhang, Yun Wang, Xinwen Chen, Rongjuan Pei

**Affiliations:** 1 State Key Laboratory of Virology and Biosafety, Wuhan Institute of Virology, Chinese Academy of Sciences 74614 , Wuhan, China; 2 University of Chinese Academy of Sciences, Beijing, China; 3 Guangzhou Laboratory 612039, Guangzhou, China; 4 Institute of Pediatrics, Guangzhou Women and Children's Medical Center, Guangzhou Medical University 26468, Guangzhou, China; The Ohio State University, Columbus, Ohio, USA

**Keywords:** SARS-CoV-2, SNARE, syntaxin-6, autophagy, virus entry

## Abstract

Despite the diminishing global impact of severe acute respiratory syndrome coronavirus 2 (SARS-CoV-2), the virus continues to circulate and undergo mutations, posing ongoing challenges for public health. A comprehensive understanding of virus entry mechanisms is crucial for managing new epidemic strains. However, the cellular processes post-endocytosis remain largely unexplored. This study employs proximity labeling to examine proteins near ACE2 post-viral infection and identified syntaxin-6 (STX6) as a factor that inhibits SARS-CoV-2 infection by impeding the endocytic release of the virus. SARS-CoV-2 infection enhances early endosome recruitment of STX6. STX6 appears to hinder the maturation of viral particles-laden early endosomes into late endosomes, from which the virus could escape. Instead, it promotes the trafficking of the virus toward the autophagy-lysosomal degradation pathway. STX6 exhibits a broad-spectrum effect against various SARS-CoV-2 variants and several other viruses that enter via endocytosis. We report for the first time the function of STX6 as a restrictive factor in viral infection.

**IMPORTANCE:**

Virus entry is the first step of the virus life cycle, and the exploitation of the endo-lysosome pathway for cellular entry by viruses has been well documented. Meanwhile, the intrinsic defense present within cells interferes with virus entry. We identified STX6 as a host restriction factor for viral entry by facilitating the virus trafficking to the autophagy-lysosomal degradation pathway. Notably, STX6 exhibits broad-spectrum antiviral activity against diverse severe acute respiratory syndrome coronavirus 2 variants and other viruses employing endocytosis for entry.

## INTRODUCTION

The coronavirus disease 2019 (COVID-19) pandemic, which was instigated by the emergence of severe acute respiratory syndrome coronavirus 2 (SARS-CoV-2), has resulted in a significant loss of human lives and considerable economic and social disruption globally (1). Despite the World Health Organization’s declaration in May 2023 that COVID-19 is no longer a public health emergency of international concern (2), SARS-CoV-2 persists in circulating and evolving globally. This situation underscores the need for continuous research across a broad spectrum, encompassing basic virology, translational medicine, and clinical studies.

Upon binding to the cell surface receptor angiotensin-converting enzyme 2 (ACE2) (3, 4), SARS-CoV-2 may enter cells via two mechanisms: membrane fusion at the plasma membrane, which necessitates the presence of the transmembrane serine protease 2 (TMPRSS2) protein, or through the endocytic pathway, where the spike protein is cleaved by cathepsin L (CTSL) to initiate membrane fusion within the late endosome/lysosome (5). It is interesting that the Omicron variant, which has been circulating since November 2021 and has led to several infection waves, is hypothesized to prefer the endocytic pathway for cell entry (6), which was supposed to affect transmission, cellular tropism, and pathogenesis (7). Numerous studies have shown that compounds targeting the endocytic entry of the virus, particularly cathepsin L inhibitors, can inhibit viral infection in cells, animals, and human tissues and may provide resistance against SARS-CoV-2 mutation escape (8–10). Additionally, host factors implicated in endocytic cargo internalization and endosomal trafficking/recycling have been identified as crucial for SARS-CoV-2 entry through several genome-wide screens (11, 12). Live cell tracking of SARS-CoV-2’s receptor-binding domain (RBD) and ACE2 has elucidated the endocytosis of RBD-ACE2, further supporting the significance of the endocytic pathway in viral entry (13).

To avoid degradation in the endo-lysosome system, viruses that enter cells through the endocytic pathway have evolved sophisticated mechanisms to release their genetic material from the endosomes (14). For instance, recent research has shown that the S protein cleavage product, S1, present in the endo-lysosome lumen, can induce endolysosome de-acidification and dysfunction through interaction with SLC38A9, thereby facilitating the escape of SARS-CoV-2 from endolysosomes (15). Conversely, cells employ a range of mechanisms to restrict viral entry. Among the host restriction factors that are particularly relevant to the endolysosome pathway for viral entry are the interferon-inducible transmembrane proteins (IFITMs) (16). IFITM3 is located on endosome vesicles that fuse with incoming viral particles and enhances the transport of vesicles containing pathogenic cargo to lysosomes, thereby limiting viral infections (17, 18). Research in mouse models has demonstrated that IFITM3 has a beneficial effect in reducing SARS‐CoV‐2 disease severity, suggesting that IFITM3 restricts the spread of SARS‐CoV‐2 (19). Additionally, the nuclear receptor coactivator 7 (NCOA7) interacts with the vacuolar H^+^-ATPase (V-ATPase) to promote vesicular acidification, lysosomal protease activity, and endocytic substance degradation. These actions impair the fusion capability of viruses adapted to the dynamics of the endo-lysosomal pathway, thus inhibiting infection. Studies have shown that NcoA7 acts as a restriction factor for SARS-CoV-2 infection (20, 21). Recently, the phospholipid scramblase 1 (PLSCR1) was identified to inhibit SARS-CoV-2 entry by preventing spike-mediated fusion, thus serving as a potent cell-autonomous restriction factor against SARS-CoV-2 infection (22, 23). Further investigation into virus-host interaction within the endosomal-lysosomal system will elucidate the intricate mechanisms at play in the initial stages of the virus-host encounter.

To elucidate the cellular response following SARS-CoV-2 infection at the stage of viral entry, we conducted a comparative proteomic analysis of ACE2 proximal proteins in the context of virus infection. This analysis led to the identification of syntaxin-6 (STX6), a member of the soluble N-ethylmaleimide-sensitive factor attachment protein receptors (SNARE), as a restriction factor for SARS-CoV-2 entry. Functionally, STX6 appears to impede the maturation of early endosomes containing viral particles into late endosomes, which are the sites from which the virus can potentially escape. Conversely, STX6 promotes the trafficking of the virus toward the autophagy-lysosomal degradation pathway.

## RESULTS

### The proximal proteome of ACE2 during SARS-CoV-2 infection

**Fig 1:**
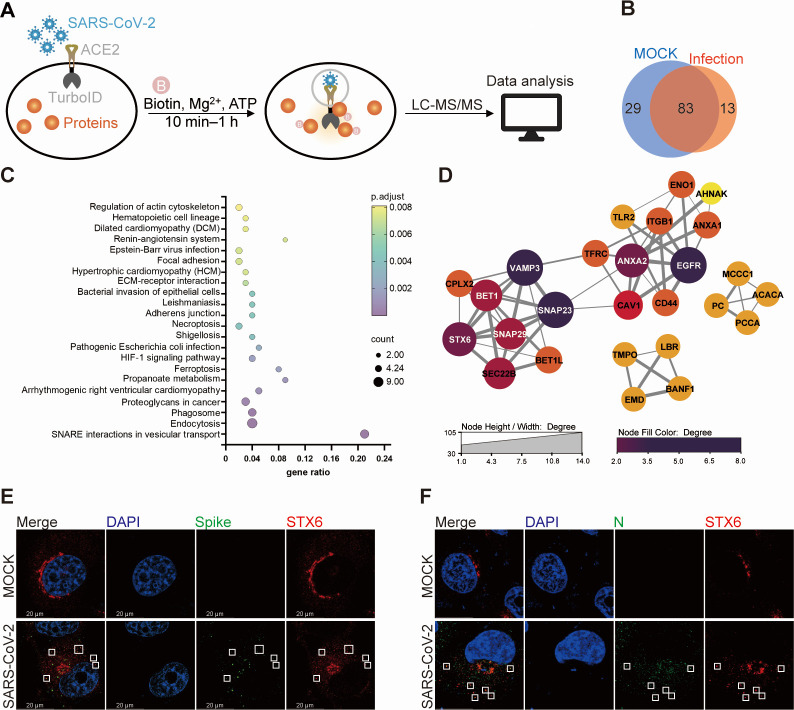
The proximal proteome of ACE2 protein during SARS-CoV-2 infection. (**A**) The schematic diagram of the study. (**B**) Venn diagrams of the ACE2 proximal proteins identified in mock and SARS-CoV-2-infected group. (**C**) The KEGG pathway enrichment analysis was performed with the ACE2 proximal proteins identified in virus-infected cells using the KOBAS 3 online tool. (**D**) The protein–protein interaction network revealed the clustering of SNARE proteins. (**E and F**) H1299-ACE2 cells were infected with SARS-CoV-2 (MOI = 50) for 1 h and fixed by paraformaldehyde. Endogenous STX6, viral Spike (**E**), and N (**F**) were stained by antibodies. The white frames indicated the colocalization of STX6 with Spike or N. Scale bars, 20 µm.

To elucidate the proteins in close proximity to ACE2 during virus infection, we genetically fused TurboID to the C-terminus of ACE2 and established a stable cell line (A549-ACE2-TurboID) expressing the fusion protein. Subsequently, A549-ACE2-TurboID cells were infected with SARS-CoV-2 at a multiplicity of infection (MOI) of 10 for 15 min with biotin supplementation in the culture medium. Biotin-labeled proteins were affinity purified using magnetic beads and subsequently identified via label-free quantitative mass spectrometry (Fig. 1A). Quantitative analysis revealed a high degree of similarity between the proteins in the mock and virus-infected groups, with AXL being the only protein identified as significantly enriched in the infected group, based on a threshold of *P.adjust* ≤ 0.05 and fold change (infection/control) ≥2. Given the capability of the TurboID enzyme to capture weak or transient interactions, we considered proteins identified in at least two out of three biological replicates in each group and excluded those likely to be contaminants according to the CRAPome database (24). A total of 112 and 96 proteins were identified as potential interactors of ACE2 in the mock and infected groups, respectively, with 13 proteins uniquely present in the infected group (Fig. 1B; Table 1). These ACE2-associated proteins in the infected group were then subjected to Kyoto Encyclopedia of Genes and Genomes (KEGG) pathway enrichment and protein interaction analysis using the KOBAS and STRING online tools (25, 26), revealing an enrichment and clustering of the SNARE interactions in vesicular transport (Fig. 1C and D). Motivated by the presence of neuropilin 1 (NRP 1), tyrosine-protein kinase receptor UFO (AXL), and epidermal growth factor receptor (EGFR) among the 13 proteins uniquely identified in the infected group, which have been established as a viral receptor or co-receptor (27–30), we selected STX6, a SNARE protein uniquely identified in the infected group, for further investigation.

**TABLE 1:** Proteins uniquely present in the infected group

Protein	Full name	Protein accession
AXL	AXL receptor tyrosine kinase	P30530.4
BANF1	Barrier to autointegration nuclear assembly factor 1	O75531.1
BET1	Bet1 Golgi vesicular membrane trafficking protein	O15155.1
CFAP45	Cilia and flagella-associated protein 45	Q9UL16.2
EGFR	Epidermal growth factor receptor	P00533.2
FAU	FAU ubiquitin like and ribosomal protein S30 fusion	P62861.2
FOLR2	Folate receptor beta	P14207.4
LBR	Lamin B receptor	Q14739.2
MLLT11	MLLT11 transcription factor 7 cofactor	Q13015.1
NRP1	Neuropilin 1	O14786.3
PCNT	Pericentrin	O95613.4
STX6	Syntaxin 6	O43752.1
VAMP3	Vesicle-associated membrane protein 3	Q15836.3

Since STX6 was identified in the ACE2 proximity proteomic analysis shortly after viral infection, we initially assessed whether STX6 interacts with ACE2 or the spike protein and whether it colocalizes with viral particles shortly after entry. Neither the spike protein nor ACE2 could be co-immunoprecipitated with STX6 (Fig. S1A and B), whereas colocalization of endogenous STX6 with SARS-CoV-2 spike and N protein was observed 1 h post-infection (Fig. 1E and F), suggesting the proximity of STX6 to endocytic viral particles.

### STX6 is a host factor restricting SARS-CoV-2 infection

**Fig 2:**
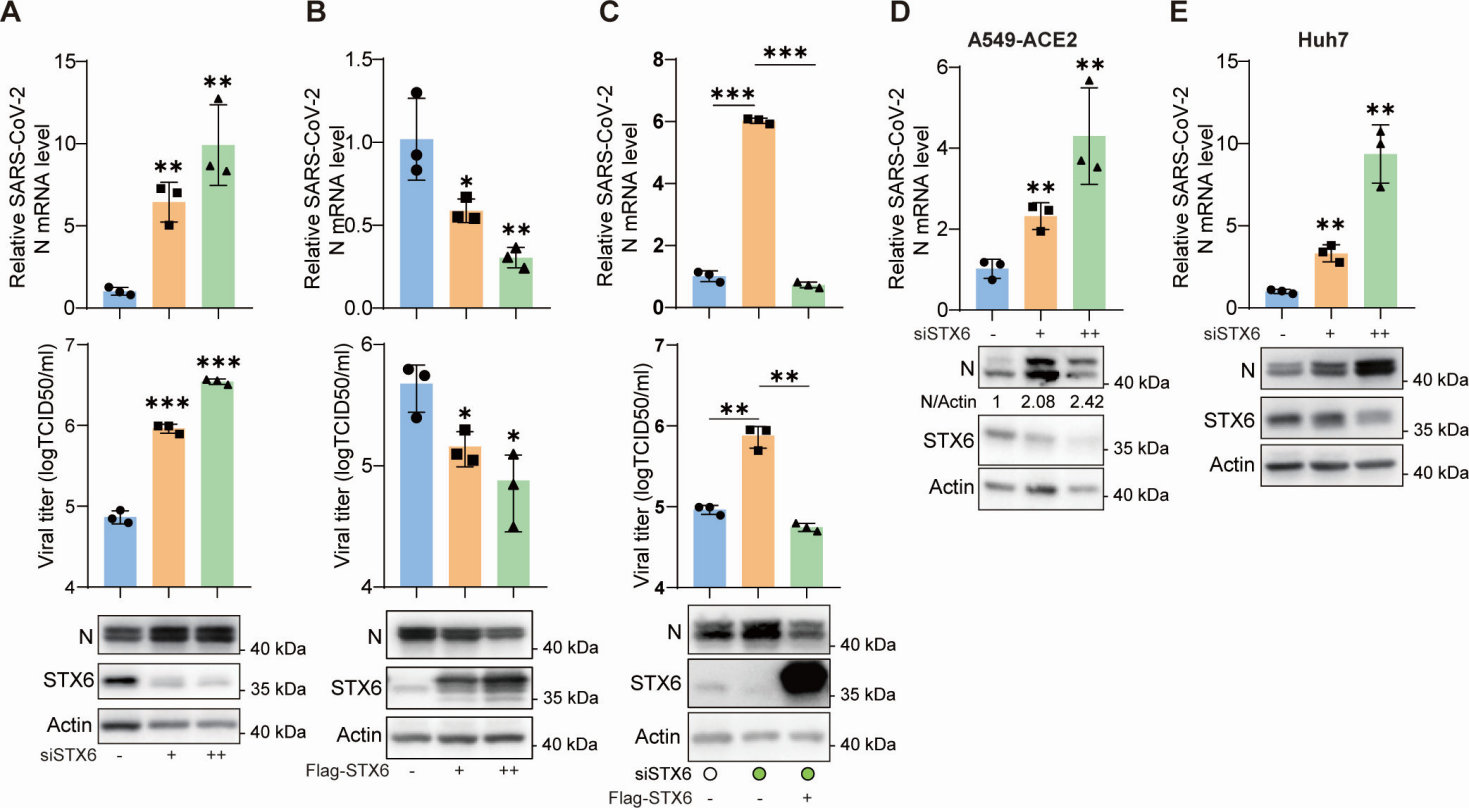
Syntaxin-6 inhibits SARS-CoV-2 infection. (**A and B**) H1299-ACE2 cells were transfected with siRNA negative control (siNC) (−), siSTX6 at 10 nM (+) or 50 nM (++) for 36 h (**A**), or transfected with vector (−), low concentration (+), or high concentration (++) Flag-STX6 plasmid for 24 h (**B**) and then infected with SARS-CoV-2 at an MOI of 0.1. The virus N mRNA level, the N protein level in cells, and the virus titer in the supernatant were quantified at 24 hpi. (**C**) H1299-ACE2 cells were first transfected with siRNAs, then complemented with vector or Flag-STX6 plasmid. Virus infection was performed at 24 h post plasmid transfection. The virus N mRNA level, N protein level in cells, and the virus titer in the supernatant were quantified at 24 hpi. (**D and E**) A549-ACE2 (**D**) and Huh7 (**E**) cells were transfected with siRNAs and then infected with SARS-CoV-2 at an MOI of 0.1. The virus N mRNA level and N protein level in cells were analyzed. The differences among groups were determined by a one-way analysis of variance followed by Tukey’s post hoc test for multiple comparisons. Data are shown as the mean ± SD. *P < 0.05, **P < 0.01, and ***P < 0.001; ns, not significant; *n* = 3.

The role of STX6 in SARS-CoV-2 infection was subsequently examined through loss-of-function and gain-of-function experiments. Knockdown of STX6 expression significantly impeded cell growth (Fig. S2A), and a stable STX6 knockout clone could not be generated using the CRISPR/Cas9 gene editing technology. Consequently, down-regulation-related studies were completed within 48 h following small interfering RNA (siRNA) treatment. After transfection of siRNA at varying concentrations, H1299-ACE2 cells were infected with SARS-CoV-2. The intracellular viral protein (N) and viral RNA level, as well as the release of infectious virus in the supernatant, were markedly increased in STX6 knockdown cells (Fig. 2A). Additional siRNAs targeting STX6 also demonstrated a pro-viral effect (Fig. S2B through D). Conversely, SARS-CoV-2 infection was attenuated in cells overexpressing STX6, as evidenced by reduced viral RNA, N protein, and virus titer (Fig. 2B). Furthermore, restoring STX6 expression via transient plasmid transfection in STX6-knockdown H1299-ACE2 cells completely abrogated the pro-viral effect induced by STX6 knockdown (Fig. 2C). The inhibitory function of STX6 on SARS-CoV-2 infection was further validated in A549-ACE2 cells and Huh7 cells, which endogenously express the ACE2 receptor (Fig. 2D and E). Collectively, these findings indicate that STX6 acts as a restriction factor for SARS-CoV-2.

### STX6 blocks SARS-CoV-2 entry

**Fig 3:**
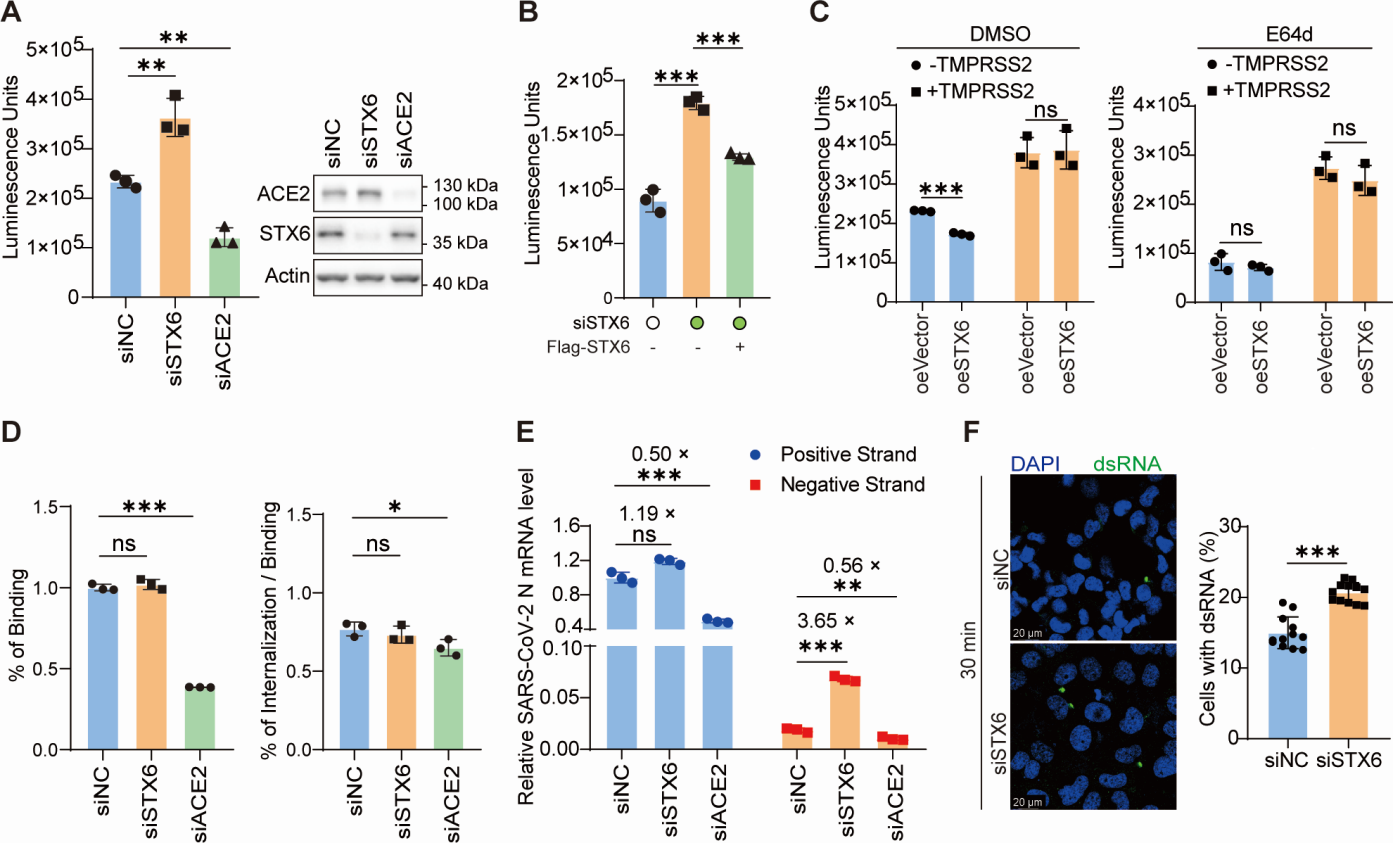
Syntaxin-6 inhibits viral invasion. (**A–C**) Luciferase activity in cell lysates was determined at 24 h post-SARS-CoV-2 spike pseudovirus transduction. (**A**) H1299-ACE2 cells were transfected with indicated siRNAs for 36 h and then transduced with SARS-CoV-2 spike pseudovirus. The knockdown efficiency was monitored by western blotting (right). (**B**) H1299-ACE2 cells were first transfected with siRNAs, then complemented with vector or Flag-STX6 plasmid, and transduced with SARS-CoV-2 spike pseudovirus. (**C**) H1299-ACE2 cells with or without ectopic expression of TMPRSS2 were transfected with Flag-STX6 plasmid, treated or untreated with E-64d (20 µM), and then transduced with SARS-CoV-2 spike pseudovirus. (**D**) H1299-ACE2 cells were transfected with indicated siRNAs, precooled, and incubated with pre-chilled SARS-CoV-2 at an MOI of 10 for 30 min at 4°C. For binding analysis, the attached virus was determined by reverse transcription real-time quantitative PCR (qRT-PCR) after removing the unbound virus. For internalization analysis, cells were transferred to 37°C and maintained for 30 min to allow internalization. Uninternalized virus particles were removed by treating the cells with 0.05% trypsin. qRT-PCR was used to detect the relative amount of internalized virus. (**E**) H1299-ACE2 cells transfected with indicated siRNA were infected by SARS-CoV-2 at an MOI of 10. SARS-CoV-2 positive- and negative-sense RNA were detected by strand-specific primers 30 min post-infection. (**F**) H1299-ACE2 cells were infected with SARS-CoV-2 at an MOI of 50, and double-stranded RNA (dsRNA) was detected with a monoclonal antibody at 30 min post-infection (left). The percentage of cells with dsRNA foci was calculated by high-content analysis (right). *n* = 12 image fields. Scale bars, 20 µm. The differences between the two groups were determined by two-tailed *t* tests. Data are shown as the mean ± SD. *P < 0.05, **P < 0.01, and ***P < 0.001; ns, not significant; *n* = 3.

Given that STX6 colocalized with SARS-CoV-2 particles shortly after infection, we proceeded to analyze the impact of STX6 on the entry efficiency of SARS-CoV-2 spike pseudovirus. Knockdown of STX6 expression increased the entry of SARS-CoV-2 spike pseudovirus, whereas restoration of STX6 expression partially mitigated the enhanced pseudovirus entry in STX6 knockdown cells (Fig. 3A and B). This suggests a role for STX6 in modulating virus entry. In cells exogenously expressing TMPRSS2, STX6 did not exhibit an inhibitory effect, indicating that STX6 does not play a role in the pathway where SARS-CoV-2 enters cells through cell surface plasma membrane fusion (Fig. 3C). Furthermore, the inhibitory effect of STX6 was abolished when the endocytic pathway of SARS-CoV-2 invasion was inhibited using the CTSL inhibitor E-64d (Fig. 3C). These results suggest that STX6 inhibits SARS-CoV-2 entry through the endocytic pathway rather than the plasma membrane fusion pathway.

Given that STX6 is implicated in protein trafficking, we assessed the expression and cell membrane localization of ACE2 using immunoblotting and flow cytometry analysis. The findings revealed that the absence of STX6 did not alter the quantity or surface distribution of ACE2 (Fig. S3A and B). Additionally, modulation of STX6 expression in H1299-ACE2 cells had no impact on the expression level of AXL, a co-receptor for SARS-CoV-2 (Fig. S3A).

We subsequently investigated whether STX6 inhibits SARS-CoV-2 binding and internalization (Fig. 3D). Virus attachment at 4°C was comparable between siNC and siSTX6 transfected H1299-ACE2 cells, whereas knockdown of ACE2 significantly diminished virus binding (Fig. 3D). The cells were then incubated at 37°C for 30 min to allow internalization of the bound virus, followed by trypsinization to remove noninternalized virus. Normalized data to negative controls revealed that STX6 knockdown did not influence virus internalization (Fig. 3D). Next, we quantified the levels of plus and minus strands RNA in the H1299-ACE2 cells 30 min post-virus infection using strand-specific reverse transcription real-time quantitative PCR (ssqRT-PCR). The results showed that in STX6 knockdown cells, the viral minus strand was significantly elevated compared to the negative control, while the viral plus strand remained largely unchanged. In ACE2 knockdown cells, both the plus and minus strands of the virus were approximately 0.5 times lower than those of the negative control group (Fig. 3E). Meanwhile, we excluded the effect of STX6 on viral RNA replication using a spike-deleted SARS-CoV-2 replicon (Fig. S3C). Based on these results, we hypothesize that STX6 does not affect viral endocytosis but rather inhibits the release of the viral genome, as only the plus-strand RNA released into the cytoplasm from endosomes can serve as templates for minus-strand RNA synthesis. Consistent with this hypothesis, the formation of double-stranded RNA (dsRNA), an intermediate product of viral replication and a surrogate marker for the entry of SARS-CoV-2 genomic RNA into the host cytosol, was increased in STX6 knockdown cells (Fig. 3F), with substantial dsRNA foci observed in approximately 20% of STX6 knockdown cells compared to about 15% of control cells at 30 min post-infection.

### Endocytosis of SARS-CoV-2 induces early endosome recruitment of STX6

**Fig 4:**
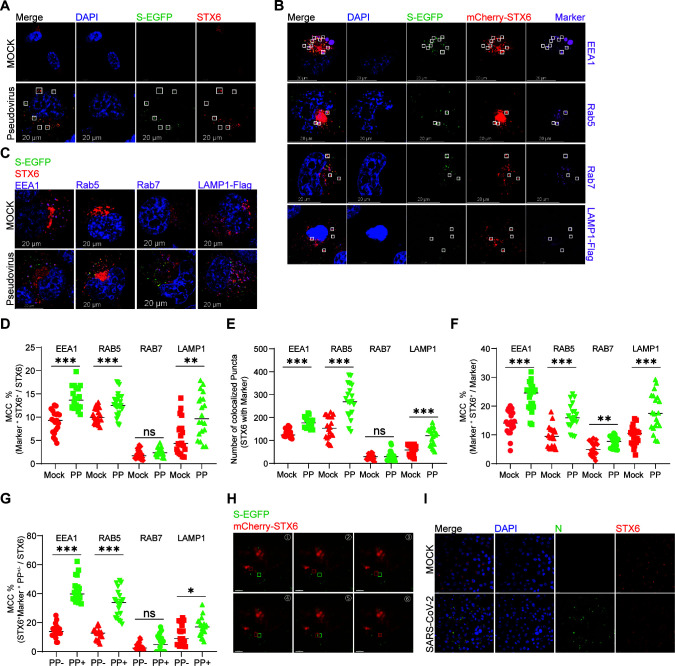
Syntaxin-6 colocalized with the virus, and both colocalized with all endosomes tested. (**A**) H1299-ACE2 cells were transduced with S-EGFP pseudovirus and fixed by paraformaldehyde at 1 h post-transduction. Endogenous STX6 was stained by antibody. The white frames indicated the colocalization of STX6 with S-EGFP. (**B**) H1299-ACE2 cells were transfected with mCherry-STX6 plasmid and then transduced with S-EGFP pseudovirus. One hour post-infection, cells were fixed, and the endogenous EEA1, Rab5, and Rab7 were stained by antibodies. The LAMP1-Flag plasmid was first transfected together with mCherry-STX6 plasmid for LAMP1-Flag staining. White frames indicate colocalization of S-EGFP, mCherry-STX6, and different markers. (**C**) Mock and S-EGFP pseudovirus transduced H1299-ACE2 cells fixed at 1 h post-infection and stained with STX6 and EEA1, Rab5, Rab7, or Flag antibodies. (**D–G**) The subcellular distribution dynamics of endogenous STX6 in Mock and S-EGFP pseudovirus transduced H1299-ACE2 cells. (**D**) The Manders’ colocalization coefficients (MCCs) represent the proportion of STX6 colocalized with different endosome markers in each cell before and after pseudovirus transduction. (**E**) The number of STX6^+^marker^+^ puncta in (**D**) was counted. (**F**) The MCCs represent the proportion of marker colocalized with STX6 in each cell before and after pseudovirus transduction. (**G**) The MCCs represent the proportion of STX6 in marker^+^PP^+^ puncta or in marker^+^PP^−^ puncta in each cell post-S-EGFP pseudovirus transduction. (**H**) Live cell fluorescence imaging shows the colocalization process of S-EGFP pseudovirus and mCherry-STX6. (**I**) Representative images showing the localization of endogenous STX6 in H1299-ACE2 cells at 24 h post-SARS-CoV-2 infection (MOI = 0.01). The differences between the two groups were determined by two-tailed *t* tests. Data are shown as the mean ± SD. *P < 0.05, **P < 0.01, and ***P < 0.001; ns, not significant; *n* = 20. Scale bars, 20 µm.

STX6 was reported to be primarily localized to the trans-Golgi network (TGN) with a small fraction found in early endosomes. Given our observation of the colocalization of endogenous STX6 with SARS-CoV-2 S and N protein shortly after infection, we proceeded to investigate the subcellular localization of STX6. For ease of manipulation and visualization, SARS-CoV-2 pseudovirus was generated using a Spike-EGFP (S-EGFP) fusion protein. Initially, we demonstrated that the cells transduced with S-EGFP pseudovirus exhibited distinct EGFP signals in punctate patterns shortly after an infection, and these EGFP signals colocalized with endogenous STX6, a finding consistent with that observed with the authentic virus (Fig. 4A). The subcellular localization of overexpressed mCherry-STX6 in various endosome compartments was subsequently analyzed by fluorescence microscopy using endocytic markers (Rab5, EEA1, Rab7, and LAMP1-Flag). One hour post-transduction, S-EGFP was detectable in early endosomes, late endosomes, and lysosomes (Fig. 4B), and colocalization of STX6 with S-EGFP was also observed in these compartments.

We further examined the subcellular distribution dynamics of endogenous STX6 following pseudovirus exposure. Consistent with observations in cells overexpressing STX6, we found that upon pseudoviral transduction, endogenous STX6 was localized to early endosomes, late endosomes, and lysosomes (Fig. 4C). Notably, the degree of colocalization between STX6 and markers EEA1 and Rab5 was significantly higher compared to its association with Rab7 and LAMP1. Moreover, pseudovirus transduction resulted in a substantial increase in the co-localization frequency of STX6 with both EEA1 and Rab5, while minimal changes were observed in the STX6-Rab7 colocalization pattern, accompanied by a slight increase in STX6-LAMP1 co-localization events (Fig. 4D and E). Specifically, approximately 14% of EEA1-positive structures displayed co-localization with STX6 in uninfected control samples, which increased to ~24% in S-EGFP pseudovirus-transduced cells. A similar trend was observed for Rab5 and LAMP1, where the fraction of Rab5-positive and LAMP1-positive structures exhibiting STX6 co-localization rose from ~9% to ~17% following pseudovirus exposure. Additionally, there was a slight increase in the co-localization rates of STX6 with Rab7 after pseudovirus challenge (Fig. 4F). We then classified the endosomal vesicles identified in pseudovirus-exposed cells into S-EGFP positive and negative and subsequently assessed STX6’s localization patterns. There was a significant increase in the percentage of S-EGFP positive EEA1/Rab5-positive compartments with colocalized STX6 compared with S-EGFP negative EEA1/Rab5-positive structures, suggesting selective recruitment of STX6 to pseudovirus-laden early endosomes (Fig. 4G).

Following the execution of live-cell real-time immunofluorescence assays, our observations indicated a gradual process of vesicular fusion, where vesicles labeled with mCherry-tagged STX6 progressively coalesced with those harboring S-EGFP pseudovirus (Fig. 4H). Additionally, the endogenous localization pattern of STX6 shifted from its characteristic perinuclear TGN distribution to a punctate distribution throughout the cytoplasm, as observed 24 h following SARS-CoV-2 infection (Fig. 4I). Together, these findings support the hypothesis that STX6 is recruited to early endosomes in the aftermath of viral infection.

### STX6 mediates the trafficking of SARS-CoV-2 to autophagic-lysosomal degradation

**Fig 5:**
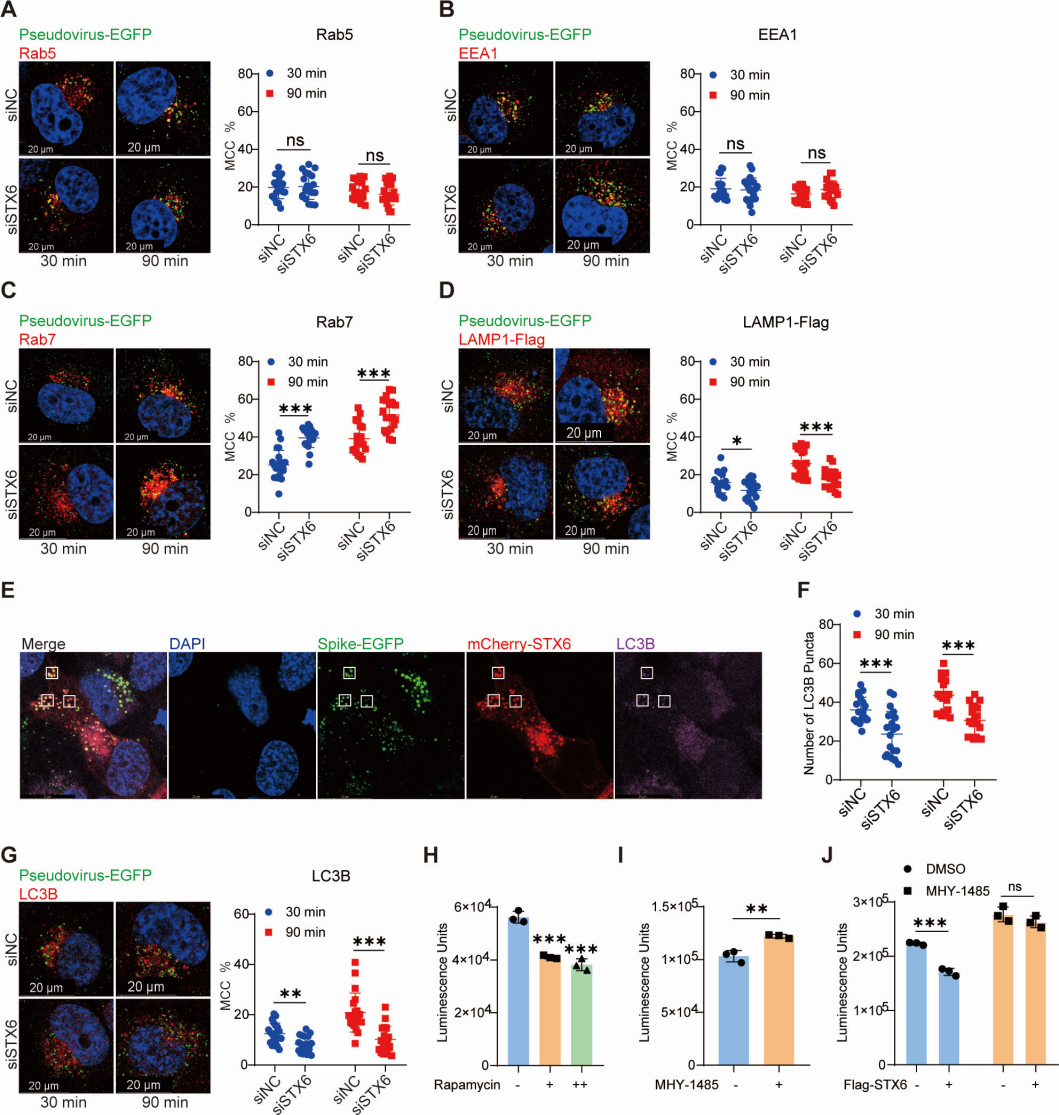
Syntaxin-6 is involved in the autophagy pathway to inhibit virus entry. (**A–D**) H1299-ACE2 cells transfected with siNC or siSTX6 were transduced with S-EGFP pseudovirus and fixed at 30 min and 90 min post-infection. Colocalization of S-EGFP pseudovirus with early endosome (left in A and B), late endosome (left in C), and lysosome (left in D) in each cell was visualized by confocal microscopy. Mander’s colocalization coefficients (MCCs) were used to calculate the proportion of S-EGFP pseudovirus colocalized with different markers (right in A–D). (**E**) H1299-ACE2 cells were transduced with S-EGFP pseudovirus after transfection of mCherry-STX6 for 24 h and were fixed at 1 hpi. The cells were subjected to immunofluorescence with antibodies against endogenous LC3B and visualized by confocal microscopy. The white frames indicated the colocalization of mCherry-STX6 with LC3B. (**F and G**) H1299-ACE2 cells transfected with siNC or siSTX6 were transduced with S-EGFP pseudovirus and fixed at 30 min and 90 min post-infection. Colocalization of S-EGFP and autophagosome (LC3B) in each cell was visualized by confocal microscopy (left in G) and calculated by MCC (right in G). The number of red puncta in (**G**), which represents LC3B puncta, was counted in F. (**H and I**) H1299-ACE2 cells were treated with 0.1 µM (+) and 1 µM (++) rapamycin (**H**) or 1 µM MHY-1485 (**I**) for 24 h, and cells were transduced with SARS-CoV-2 spike pseudovirus for 24 h. Luciferase activity in cell lysates was determined. (**J**) H1299-ACE2 cells overexpressing vector or Flag-STX6 plasmid were treated with dimethyl sulfoxide (DMSO) and MHY-1485 (1 µM) for 24 h. Cells were transduced with SARS-CoV-2 pseudovirus for 24 h. Luciferase activity in cell lysates was determined. The differences between the two groups were determined by a two-tailed *t* test. Data are shown as the mean ± SD. *P < 0.05, **P < 0.01, and ***P < 0.001; ns, not significant; *n* = 20 (**A–D, F, and G**), *n* = 3 (**H–J**). Scale bars, 20 µm.

To elucidate the cellular mechanisms underlying STX6’s inhibition of viral entry, we assessed the effect of STX6 on the distribution of endocytosed virion with various endocytic vesicles in control and STX6 knockdown cells. Silencing of STX6 expression did not influence the colocalization of pseudovirus with Rab5 and EEA1 (Fig. 5A and B). However, the colocalization of pseudovirus with Rab7 was markedly elevated in STX6 knockdown cells at both 30 and 90 min post-infection (Fig. 5C), whereas its colocalization with LAMP1 was modestly reduced at the same time points (Fig. 5D). These findings, in conjunction with the recruitment of STX6 to early endosomes upon infection, imply that STX6 does not affect the initial entry of SARS-CoV-2 into early endosomes but may modulate the maturation of virion containing early endosomes into late endosomes.

Considering that STX6 possesses two LC3-interacting regions and interacts with LC3B (31), a marker for autophagosomes, we evaluated the relationship between STX6 and auto-lysosomal degradation. Indeed, the colocalization of S-EGFP pseudovirus and mCherry-STX6 with autophagosomes, as indicated by LC3B puncta, was observed (Fig. 5E). Additionally, the number of LC3B puncta was markedly diminished in pseudovirus-transduced cells when STX6 was knocked down (Fig. 5F), and the proportion of virus co-localization with autophagosomes was also reduced (Fig. 5G). These findings suggest that STX6 contributes to the formation of autophagosomes in response to pseudovirus entry. An autophagy activator rapamycin significantly inhibited pseudovirus invasion (Fig. 5H), while an autophagy inhibitor, MHY-1485, modestly promoted the pseudovirus invasion and counteracted the inhibitory effect of STX6 (Fig. 5I and J). In conclusion, STX6 functions as a host defense protein that facilitates an alternative degradation pathway for endocytosed viruses.

### Proper localization of STX6 is required in the viral restriction function

**Fig 6:**
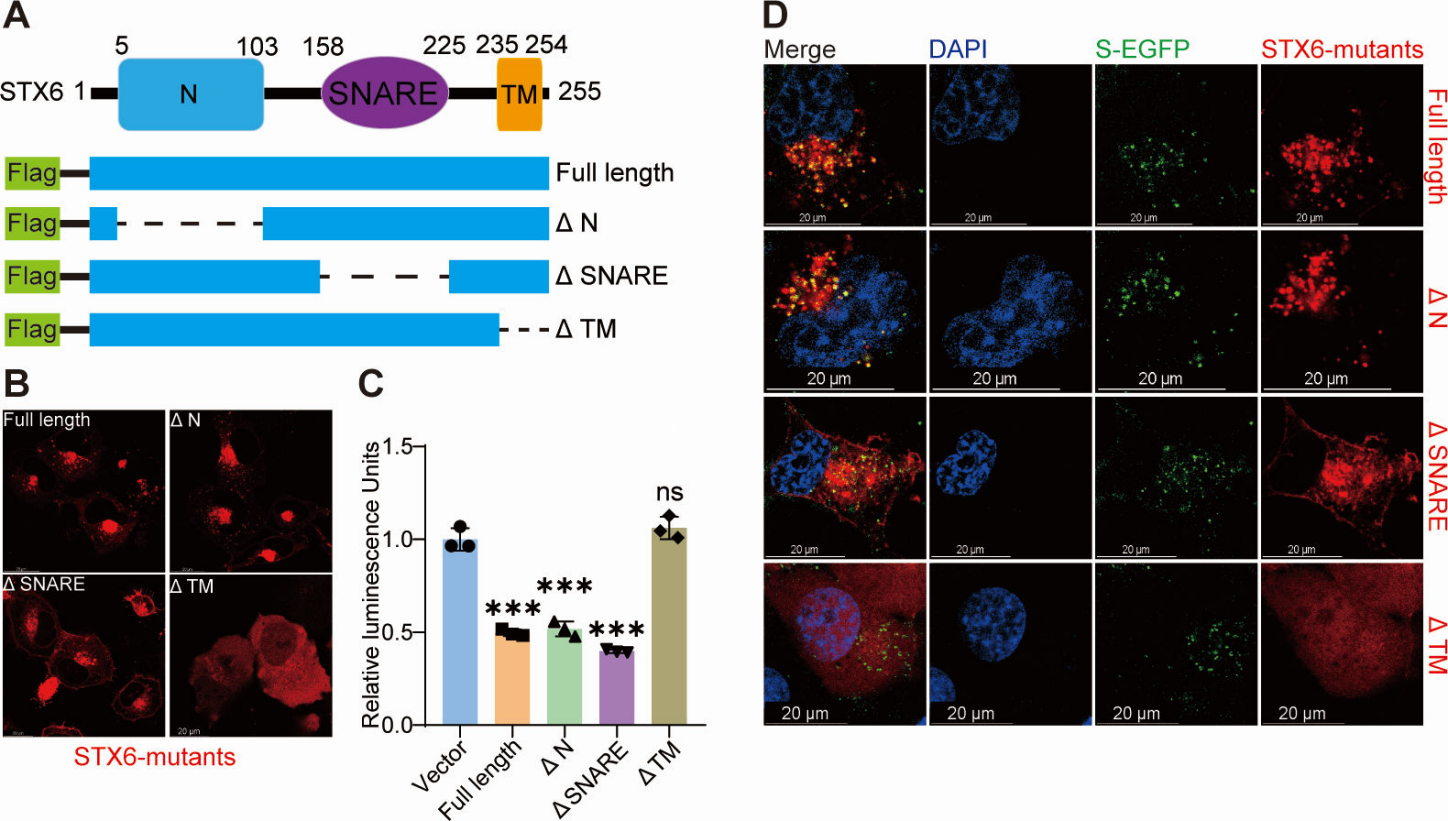
Proper localization of Syntaxin-6 is required in the viral restriction function. (**A**) Schematic diagram depicting the deletion mutants of STX6: full length (1–255), Δ N (delete 5–103), Δ SNARE (delete 158–225), and Δ TM (1–234). (**B**) Representative images showing the localization of full length and deletion mutants of STX6. (**C**) H1299-ACE2 cells were transfected with full length or deletion mutants of STX6 for 24 h, and cells were transduced with SARS-CoV-2 spike pseudovirus for 24 h. Luciferase activity in cell lysates was determined. (**D**) Representative images showing the localization of full length or deletion mutants of STX6 as well as S-EGFP in H1299-ACE2 cells at 1 h post-S-EGFP pseudovirus transduction. The differences among groups were determined by a one-way analysis of variance followed by Tukey’s post hoc test. Data are shown as the mean ± SD. *P < 0.05, **P < 0.01, and ***P < 0.001; ns, not significant. *n* = 3. Scale bars, 20 µm.

Drawing on the known functional domains, we generated a series of mutants (Fig. 6A) to investigate the correlation between the structure and function of STX6. The deletion of the C-terminal transmembrane domain (Δ TM) resulted in a uniform cellular distribution of STX6, with no colocalization observed with the pseudovirus (Fig. 6B and D). This mutant also forfeited its capacity to inhibit virus entry (Fig. 6C). The STX6 mutant lacking the N-terminal domain (Δ N) displayed no alteration in subcellular localization relative to the full-length protein, continued to exhibit colocalization with the pseudovirus, and preserved its ability to inhibit pseudovirus entry (Fig. 6B through D). After the deletion of the SNARE motif (Δ SNARE), STX6 exhibited a distinct plasma membrane localization yet sustained colocalization with the pseudovirus and maintained the capacity to inhibit pseudovirus entry (Fig. 6B through D). Therefore, the N and SNARE domains are sufficient but not obligatory for STX6’s inhibitory effect on SARS-CoV-2 invasion, whereas the membrane localization of STX6 is crucial.

### STX6 has pan-antiviral activity

**Fig 7:**
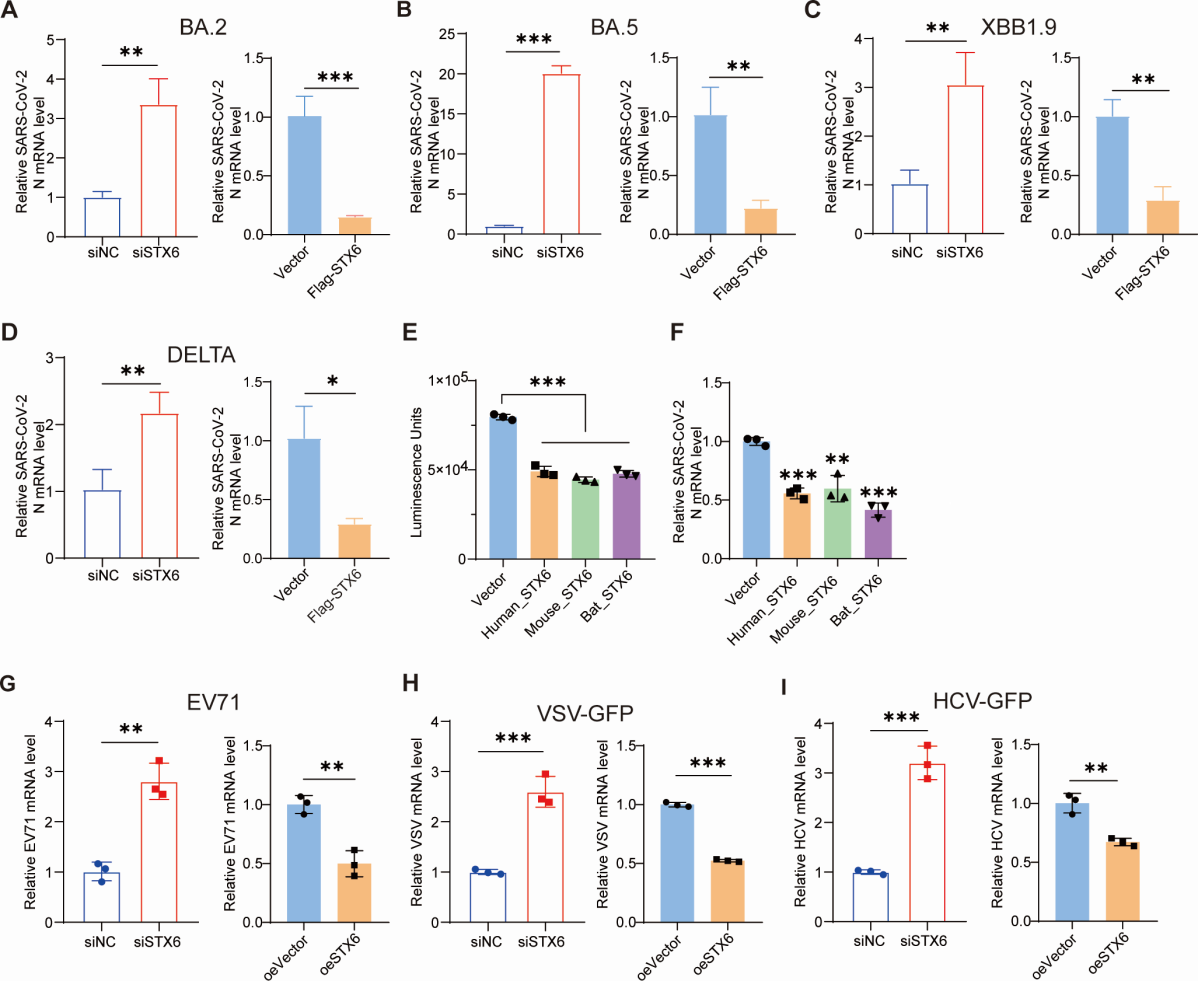
Syntaxin-6 has antiviral activity against variants of concern (VOCs) and other endocytic intrusion viruses. (**A–D**) H1299-ACE2 cells were transfected with control siNC, siSTX6 for 36 h (left in A–D), or transfected with vector, flag-STX6 plasmid for 24 h (right in A–D) and then infected with BA.2 strain (**A**), BA.5 strain (**B**), XBB1.9 strain (**C**), and delta strain (**D**) at an MOI of 0.1. The virus N mRNA level was quantified at 24 hpi. (**E and F**) SARS-CoV-2 pseudovirus transduction (**E**) and SARS-CoV-2 infection (**F**) in H1299-ACE2 cells transfected with vector or human (*Homo sapiens*), mouse (*Mus musculus*), or bat (*Rhinolophus sinicus*) orthologs of STX6. (**G–I**) Hela (**G**), H1299-ACE2 (**H**), or Huh7.5.1 (**I**) cells were transfected with control siNC, siSTX6 for 36 h (left), or transfected with vector, flag-STX6 plasmid for 24 h (right) and then infected with EV71 (**G**), VSV-GFP (**H**), or HCV J399EM (**I**), respectively, at an MOI of 0.1. The virus RNA level was determined by qRT-PCR. The differences between the two groups were determined by two-tailed *t* test, except e and f (one-way analysis of variance followed by Tukey’s post hoc test). Data are shown as the mean ± SD. *P < 0.05, **P < 0.01, and ***P < 0.001; ns, not significant; *n* = 3.

To ascertain whether STX6 limits the infection of various SARS-CoV-2 variants, we conducted further experiments to assess the impact of STX6 knockdown and overexpression on SARS-CoV-2 delta and Omicron BA.2, BA.5, and XBB.1.9 strains. An increase in viral RNA levels was observed in STX6 knockdown cells, whereas a decrease was noted in STX6 overexpression cells (Fig. 7A through D). These observations suggest that STX6 exhibits broad antiviral activity against multiple SARS-CoV-2 variants. This wide-ranging antiviral profile led us to investigate whether such activity is evolutionarily conserved. Given that bats are known to be natural reservoirs for SARS-like coronaviruses (32), we hypothesize that STX6 may provide anti-SARS-CoV-2 activity in bats and in experimental models such as mice. Through protein analysis using multiple sequence alignment, we identified that the two key domains of STX6 (N-terminal domain and SNARE motif) are highly conserved across *Homo sapiens* (NP_005810.1), *Mus musculus* (NM_021433.3), and *Rhinolophus sinicus* (XM_019734272.1; Fig. S4A). Notably, overexpression of cross-species STX6 significantly inhibited the entry of SARS-CoV-2 pseudovirus and reduced the viral RNA level in SARS-CoV-2-infected cells (Fig. 7E and F). Therefore, human, mouse, and bat STX6 orthologs can potentially be functionally interchanged to confer resistance to SARS-CoV-2.

Furthermore, we evaluated the antiviral activity of STX6 against a panel of other viruses that enter cells via endocytosis, including HCoV-OC43, HCoV-229E, enterovirus A71 (EV71), vesicular stomatitis virus (VSV), and hepatitis C virus (HCV). The impact of STX6 on HCoV-OC43 and HCoV-229E infections was marginal (Fig. S4B and C), whereas STX6 significantly inhibited EV71, VSV, and HCV infections (Fig. 7G through I; Fig. S4D). In conclusion, STX6 exhibits a broad-spectrum effect against various SARS-CoV-2 variants and several other viruses that enter via endocytosis.

## DISCUSSION

By conducting a comprehensive analysis of the proteomic changes in the vicinity of ACE2 during SARS-CoV-2 infection, we have identified STX6 as a key host restriction factor that impedes viral infection. Our data indicate that STX6 is recruited to early endosomes following SARS-CoV-2 infection. This recruitment appears to inhibit the maturation of early endosomes into late endosomes while simultaneously enhancing autophagosome formation. Consequently, these autophagosomes promote the degradation of viral particles through the autophagy-lysosomal pathway, thereby limiting viral replication and spread.

It is widely recognized that viruses have evolved complex strategies to evade cellular antiviral defenses. A well-documented mechanism involves the exploitation of the endo-lysosome pathway for cellular entry by viruses. Simultaneously, host cells have developed intrinsic immunity factors, such as IFITMs, NCOA7, LY6E, and PLSCR1, which counteract viral invasion by promoting virion degradation within the endo-lysosome system (22, 23, 33). Our discovery that STX6 is recruited to early endosomes containing incoming virions and promotes autophagosome formation reveals a novel mechanism of intrinsic immunity during the initial stages of SARS-CoV-2 infection. The interactions between SARS-CoV-2 replication and the autophagy-lysosome pathway have been extensively studied. While prevailing evidence suggests a pro-viral role for the autophagy machinery (34), the observation that SARS-CoV-2 employs multiple mechanisms to counteract autophagy highlights the potential antiviral function of this pathway (35–37). Our experimental data demonstrate that inhibition of the autophagy-lysosome pathway enhances the entry of SARS-CoV-2 pseudovirus, whereas stimulation of autophagy through mTOR inhibitor reduces it, thereby providing compelling evidence for the antiviral function of the autophagy-lysosome pathway during the viral entry phase.

As a key SNARE protein, STX6 is mainly localized within the trans-Golgi network and early endosomes, where it participates in various fusion events, including retrograde transport, endosome-to-Golgi trafficking, and vesicular fusion (38, 39). Research from Prof. Hong Zhang’s laboratory (40) has demonstrated that *O*-linked β-*N*-acetylglucosamine (*O*-GlcNAc) transferase (OGT) mediates *O*-GlcNAcylation of the SNARE protein SNAP-29, modulating autophagy in a nutrient-dependent manner. The researchers hypothesized that augmented formation of the SNAP-29/Stx6 SNARE complex may elevate autophagy activity in *OGT* knockdown cells, potentially via its role in endocytic trafficking. In hepatocellular carcinoma cells, STX6 has been reported to promote autophagy flux under nutrient repletion conditions likely by facilitating the association of autophagosomes with lysosomes (41). In Beclin 2-mediated non-canonical autophagy, STX5 and STX6 interact with Beclin 2 to promote the fusion of MEKK3- or TAK1-associated ATG9A^+^ vesicles with phagophores for subsequent degradation (42). Our findings revealed a decrease in LC3 puncta formation upon STX6 knockdown in SARS-CoV-2 pseudovirus-transduced cells, suggesting a potential role for STX6 in autophagosome formation. Similarly, Takashi Nozawa et al. found that STX6 forms complexes with VAMP3 and VTI1B to mediate the fusion of autophagosomes and recycle endosomes during bacterial infection (43). However, further research is necessary to elucidate the precise function of STX6 in the stepwise process of autophagosome maturation, such as precursor generation, assembly, and expansion. Additionally, some studies suggest a collaborative involvement of RAB proteins, tethering proteins, and the SNARE complex in the fusion process between autophagosomes and various stages of endosomes. Further exploration is warranted to identify other proteins that may function in conjunction with STX6.

Although it is widely acknowledged that autophagy serves as an intrinsic antiviral mechanism for the elimination and degradation of invading microorganisms, the initiation of autophagosome biogenesis in mammalian cells in response to viral infection remains poorly understood. SARS-CoV-2 may induce autophagy during entry, as both SARS-CoV-2 spike pseudovirus and recombinant SARS-CoV-2 spike protein treatment have been shown to elicit autophagic responses in ACE2-expressing cells (44). Jacqueline Staring (45) demonstrated that the danger receptor galectin-8 can detect endosome damage during picornavirus infection and mediate the autophagic clearance, whereas the lipid-modifying enzyme PLA2G16 facilitates the release of viral genome from the endosome and prevents clearance. SNX5 has been shown to interact with Beclin 1 and ATG14-containing class III phosphatidylinositol-3-kinase (PI3KC3) complex 1 (PI3KC3-C1) and is essential for the generation of autophagosome at the virion-containing endosomes (46). Further elucidation of how STX6 is recruited to early endosomes and whether it functions downstream of the damage sensor galectin-8 or the virophage-specific mediator SNX5 would be of considerable interest.

Our study has demonstrated that viral infection induces a redistribution of endogenous STX6, shifting its primary perinuclear TGN location to a more punctate distribution in the cytosol. This alteration is hypothesized to be triggered by the virus, as our data indicate that pseudovirus invasion leads to an increased presence of STX6 in early endosomes. Furthermore, during the replication stage, SARS-CoV-2 may induce the translocation of STX6, as ORF3a has been shown to increase the formation of STX6 puncta within cells (47). ORF3a also results in the activation of Rab7, which inhibits endolysosome formation and disrupts lysosomal hydrolase transport (48). Consequently, this intriguing discovery suggests that STX6 may play a pivotal role in the viral invasion process.

Our findings indicate that STX6 exerts a broad antiviral function by restricting several variants of SARS-CoV-2. However, its role becomes more complex when considering other endocytosed viruses. Several factors merit further investigation. First, the mechanism underlying STX6 to early endosomes remains unclear; it is unknown whether this recruitment depends on viral factors or virus-triggered signaling. Several factors warrant further investigation. Second, the induction of autophagy and the counteraction of the virus need consideration. Consistent with previous findings that VSV-G and HCV envelope proteins may initiate autophagy (49, 50), and EV71 can trigger autophagy by pore formation (45), our results suggest a restriction role of STX6 in these viruses’ infection. Additionally, the broad antiviral function of STX6 requires further investigation. Existing reports suggest that SNARE proteins may be involved in virus replication. For instance, previous reports have indicated that STX6 is necessary for the trafficking of human cytomegalovirus to the TGN in monocytes (51).

The emergence of proximity labeling technology has enabled the identification of novel protein–protein interactions (PPIs) and the mapping of subcellular proteomes. Our study was initially designed to delineate the immediate environment of the viral receptor during infection. The lack of substantial alterations in proteomic profiles aligns with expectations, as the processes of virus binding and internalization are not expected to induce significant membrane modifications. Utilizing the sensitivity of turboID technology in detecting transient and weak interactions, we have identified several proteins that are uniquely present in the virus-infected group. This includes known receptors or co-receptors implicated in SARS-CoV-2 entry, such as NRP1, AXL, and EGFR. Additionally, along with STX6, two SNARE proteins were detected. However, the assembly of these SNARE proteins into a functional SNARE complex requires further analysis.

Our research findings highlight the essential role of STX6 in orchestrating the intracellular trafficking of internalized viruses toward auto-lysosomal degradation, a critical step during the viral entry process. Specifically, our data indicate that STX6 serves as a key component of the cell’s innate immune defense mechanisms, exhibiting potential to suppress the proliferation of SARS-CoV-2 variants of concern.

## MATERIALS AND METHODS

### Cell lines, viruses, and biosafety

Vero E6 (American Type Culture Collection [ATCC], CRL-1586), HEK-293T (ATCC, CRL-11268), A549 (ATCC, CCL-185), HCT-8 (ATCC, CCL-244), HeLa (CCL-2), and Huh7.5.1 (kindly provided by Prof. Jin Zhong) cells were cultured in Dulbecco’s modified Eagle medium (DMEM, Gibco) supplemented with 10% heat-inactivated fetal bovine serum (FBS) and 1% penicillin-streptomycin. A549-ACE2-TurboID cells were generated by stably expressing hACE2-TurboID in the aforementioned original cell lines using a lentiviral system. NCI-H1299-ACE2 cells (kindly provided by Prof. Jianhua Zhang) were cultured in Roswell Park Memorial Institute 1640 (RPMI, Gibco) with 10% FBS and 1% pen-strep. All cells were regularly tested for mycoplasma contamination and authenticated by visual observations of cell morphology and were maintained at 37°C in a fully humidified atmosphere containing 5% CO_2_ unless otherwise stated.

SARS-CoV-2 wild type (CSTR: 16533.06.IVCAS6.7512) (3), delta variant strain B.1.617.2 (CSTR: 16533.06.IVCAS 6.7585, isolated by Institute of Laboratory Animal Science, CASM& PUMC), Omicron BA.2 strain (CSTR: 16533.06.IVCAS 6.7617, isolated by Hubei Center for Disease Control and Prevention), Omicron BA.5 strain (CSTR: 16533.06.IVCAS 6.8981, isolated by National Institute for Viral Disease Control and Prevention, China CDC), and Omicron XBB.1.9.1 strain (CSTR: 16533.06.IVCAS 6.9084) were obtained from the National Virus Resource Center (Wuhan, China). The above virus stocks were prepared by virus amplification in Vero-E6 cells. The hepatitis C virus (J399EM) was prepared as previously described (52). Vesicular stomatitis virus-EGFP (VSV-EGFP) was a gift from prof. Hualan Chen. Enterovirus A71 was kindly provided by Dr. Yuzhi Fu. HCoV-229E (VR-740) and HCoV-OC43 (VR-1558) were brought from ATCC and were amplified in Huh7 cells and HCT-8 cells.

All experiments with live SARS-CoV-2 viruses were carried out in the Biosafety Level 3 (BSL-3) laboratory at Wuhan National Biosafety Laboratory.

### Antibodies and reagents

The following antibodies were used in this study: Rabbit anti-syntaxin 6 polyclonal antibody (10841–1-AP), rabbit anti-ACE2 polyclonal antibody (21115–1-AP), rabbit anti-AXL polyclonal antibody (13196–1-AP), mouse anti-TFRC monoclonal antibody (66180–1-Ig), mouse anti-Beta Actin monoclonal antibody (66009–1-Ig), mouse anti-GAPDH monoclonal antibody (60004–1-Ig), mouse anti-EEA1 monoclonal antibody (68065–1-Ig), rabbit anti-Rab7A polyclonal antibody (55469–1-AP), and mouse anti-LAMP1 monoclonal antibody (67300–1-Ig) were obtained from Proteintech. Mouse anti-Rab5A monoclonal antibody (46449S), rabbit anti-HA-Tag monoclonal antibody (3724S), rabbit anti-FLAG antibody (2368S), and mouse anti-dsRNA monoclonal antibody (76651L) were obtained from Cell Signaling. Rabbit anti-spike polyclonal antibody (40589-T62) and mouse anti-nucleocapsid monoclonal antibody (40143-MM05) were obtained from Sino Biological. Rabbit anti-LC3b monoclonal antibody (A19665) was purchased from ABclonal. Peroxidase AffiniPure goat anti-mouse IgG (H + L; 115-035-146) and Peroxidase AffiniPure goat anti-rabbit IgG (H + L; 111-035-003) were purchased from Jackson ImmunoResearch. Goat anti-rabbit IgG (H + L) cross-adsorbed secondary antibody 633 (A21070), goat anti-mouse IgG (H + L) cross-adsorbed secondary antibody 633 (A21050), donkey anti-rabbit IgG (H + L) highly cross-adsorbed secondary antibody 568 (A10042), donkey anti-mouse IgG (H + L) highly cross-adsorbed secondary antibody 568 (A10037), donkey anti-rabbit IgG (H + L) highly cross-adsorbed secondary antibody 488 (A21206), and donkey anti-mouse IgG (H + L) highly cross-adsorbed secondary antibody 488 (A21202) were obtained from Invitrogen. Chemicals and reagents are as follows: Lipofectamine RNAiMAX (13778–150), Lipofectamine 3000 (L3000-015), and Hoechst 33258 (H1398) were purchased from Invitrogen. Aloxistatin (E64d, HY-100229), MHY1485 (HY-B0795), and Rapamycin (HY-10219) were obtained from MedChemExpress (MCE).

### Sample preparation for proteomics experiment

For each sample, A549 cells with stably expressing ACE2-TurboID were grown as a monolayer in a 10 cm dish. The cells at ~80% confluency were infected with SARS-CoV-2 for 15 min at an MOI of 2, and at the same time, TurboID samples were labeled using 500 µM biotin, 1 mM ATP, and 5 mM MgCl2 for 15 min. Labeling was stopped by placing cells on ice and washing them six times with ice-cold phosphate buffered saline (PBS). Cells were detached in ~1.5 mL radioimmunoprecipitation assay (RIPA) lysis buffer (50 mM Tris pH 8, 150 mM NaCl, 0.1% SDS, 0.5% sodium deoxycholate, 1% Triton X-100, protease inhibitor cocktail [Roche], and 1 mM phenylmethylsulfonyl fluoride [PMSF]) from the flask using a cell scraper and then incubated for 30 min on ice. Lysates were clarified by centrifugation at 12,000 × *g* for 10 min at 4°C.

To enrich biotinylated material from proteomic samples, 200 µL streptavidin-coated magnetic beads (Pierce) were washed twice with RIPA buffer, incubated with clarified lysates for each sample with rotation for 1 h at room temperature, then moved to 4°C, and incubated overnight with rotation. The beads were subsequently washed twice with 1 mL of RIPA lysis buffer, once with 1 mL of 1 M KCl, once with 1 mL of 0.1 M Na_2_CO_3_, once with 1 mL of 2 M urea in 10 mM Tris-HCl (pH 8.0), and twice with 1 mL RIPA lysis buffer. The beads were then shipped to BioSpec on ice for further processing and preparation for liquid chromatography-tandem mass spectrometry (LC-MS/MS) analysis.

### PPI network

The PPIs among the proteins that appeared in the infected group were evaluated using the STRING database (https://string-db.org/) (26). Validated interactions with a combined score >0.9 were selected as significant. Then, the integration of PPI networks was constructed using the Cytoscape software (V3.10.3; https://cytoscape.org) (53), where the node size and color intensity represent the degree of the node, and the edge thickness reflects the combined score. Nodes with a degree ≥3 were retained for further analysis.

### Tissue culture infectious dose assays

Tissue culture infectious dose (TCID_50_) assays were performed on VeroE6 cells. Briefly, VeroE6 monolayers were grown to 80% confluency in 96-well plates and incubated for 1 h at 37°C in 5% CO_2_ with serially diluted virus supernatants. The cells were incubated in DMEM supplemented with 2% FBS. Cells (37°C, 5% CO_2_, and 95% relative humidity) for 4 d. The cytopathic effect was observed under the microscope, and the TCID_50_ was calculated with the Reed–Muench method.

### Cloning

The genetic constructs used in this study were listed in Table S1, with a detailed description of construct designs, linker orientations, and epitope tags. For cloning, PCR fragments were amplified using Transtart FastPfu DNA polymerase (TransGen Biotech). The vectors were double-digested using standard enzymatic restriction digest and ligated to gel-purified PCR products by T4 DNA ligation. Ligated plasmid products were introduced by heat shock transformation into competent DH5α.

### Lentivirus production

Delivery of expression plasmids for overexpression experiments was done through transduction with lentiviruses. For preparation of lentivirus stocks, HEK-293T cells in T25 flasks were transfected at ~90% confluency with packaging plasmids psPAX2 (Addgene, 2,250 ng) and pMD2.G (Addgene, 750 ng) and specific expression plasmids (3,000 ng, Table S1). After 60 h, the media was removed and centrifuged at 5,000 × *g* at 4°C for 10 min to pellet cell debris. The supernatant was filtered through a 0.45 µm low protein-binding membrane (Millipore). For all experiments, transductions were performed in the presence of 10 µg mL^−1^ polybrene.

### RNA interference experiments

The siRNA duplexes specific to different genes were purchased from GenePharma. A list of siRNA sequences is described in Table S2. The siRNA duplexes were transfected using Lipofectamine RNAiMAX reagent (Invitrogen) at a final concentration of 50 nM or 10 nM according to the manufacturer’s instructions. Nonsilencing siRNA with a scrambled sequence was used as a negative control (siNC).

### Pseudovirus production and transduction

One day before transfection, HEK-293T cells were plated into 10 cm culture dishes and maintained in 10 mL DMEM containing 10% FBS. When the cell confluence was 80%, HEK-293T cells were co-transfected with 8 µg of pNL4.3-luc-R-E- and 8 µg of SARS-CoV-2 SpikeΔC1-19 plasmids, and the transfection operation was performed according to the instructions for Lipo3000. The virus was harvested 48 h after transfection and centrifuged at 3,000 r/min for 10 min to remove cell debris, and the supernatant was carefully aspirated and filtered through a 0.45 µm filter, packaged, and stored at −80°C for further use. When used in confocal experiments, spike ΔC1-19 was replaced by spike ΔC1-19-EGFP. At the same time, the supernatant was concentrated with 8% PEG8000 and resuspended with RPMI 1640 cell culture medium.

For the pseudovirus transduction assay, H1299-ACE2 cells were seeded in a 24-well plate. After transfection, 200 µL pseudovirus was added to each well. The plate was incubated at 37°C for 2 h and then replaced with fresh medium to maintain the culture. Pseudovirus entry in cells was assessed 24 h later by measuring firefly luciferase expression.

### Virus binding and internalization assay

H1299-ACE2 cells were precooled at 4°C for 30 min and then incubated with pre-chilled SARS-CoV-2 (MOI = 10) for 30 min at 4°C. Unbound virus particles were removed by washing three times with pre-chilled PBS. Relative amounts of bound virus were determined by qRT-PCR. For viral internalization assays, cells were incubated with SARS-CoV-2 using the same conditions described above. Cells were then transferred to 37°C and maintained for 30 min to allow internalization of the bound virus. Uninternalized virus particles were removed by treating the cells with 0.05% trypsin. Another group of cells was trypsinized directly after virus binding to digest all bound but not internalized virus as a negative control. qRT-PCR was used to detect the relative amount of internalized virus.

### Western blotting and Co-immunoprecipitation

Whole-cell lysates were obtained by lysis of cells for 30 min on ice with cell lysis buffer for western and IP (P0013) adding PMSF (ST506) purchased from Beyotime. Cell lysates were centrifuged at 14,000 × *g* for 10 min at 4°C. For western blotting (WB), the supernatants were recovered and followed by denaturation at 95°C for 10 minutes. For co-immunoprecipitation, the supernatants were collected and mixed with Protein G-agarose (Millipore) and various antibodies for 16 h at 4°C. After six washes using ice-cold lysis buffer, Protein G agarose-bound immune complexes were then eluted and subjected to WB analysis. Each sample was resolved by SDS-PAGE and transferred to nitrocellulose. Membranes were blocked with Tris buffered saline with Tween (TBST) (pH 7.4, containing 0.1% Tween-20) containing 5% skimmed milk for 1 h at room temperature, followed by incubation with anti-sera containing primary antibodies overnight at 4°C. Membranes were washed and incubated for 1 h at room temperature with the horseradish peroxidase (HRP)-conjugated secondary antibodies. Membranes were imaged using the FluorChem HD2 system (Alpha Innotech). Images were analyzed using AlphaEaseFC software (Alpha Innotech).

### Reverse transcription real-time quantitative PCR and ssqRT-PCR

Total cellular RNA was isolated with TRIzol (Invitrogen) reagent according to the manufacturer’s protocols. Viral RNA in culture supernatants was extracted using a QIAamp Viral RNA Mini Kit (Qiagen) according to the manufacturer’s instructions. The quantification of specific gene transcripts was analyzed by one-step real-time qRT-PCR with the HiScript II One Step qRT-PCR SYBR Green Kit (Vazyme) using specific primers and the Applied Biosystems QuantStudio 6 Flex. The primer sequences for qRT-PCR were designed using Primer5 software (see Table S3). The data were normalized to levels of β-actin mRNA in each individual sample. For all experiments, the 2−ΔΔCt method and standard curve line were used to calculate relative expression changes and absolute quantification, respectively. For ssqRT-PCR, RNA extraction was performed exactly as described above. In brief, non-viral gene sequence tags were added to the 5′ end of the plus and minus strand-specific reverse transcription primers of the viral N protein for reverse transcription. Then qRT-PCR was performed with specific primers. Primer sequences used for ssqRT-PCR are shown in Table S3.

### Immunofluorescent confocal microscopy and colocalization analysis

Cells cultured on confocal glass dishes were washed with PBS and then fixed with 4% paraformaldehyde at room temperature for 20 min and permeabilized in 0.5% Triton X-100 for 10 min at 4°C. Coverslips were blocked in 1% normal goat serum (AR1009, Boster) in PBS for 1 h. Samples were incubated with primary antibodies (1:200 diluted) overnight at 4°C followed by incubation with secondary antibodies (1:500 diluted) for 1 h at 37°C. Then dishes were washed with PBS and stained with Hoechst 33258 for 10 min at room temperature, followed by observing using a Leica STELLARIS 8 WILL confocal microscope under a 63× oil objective. The cells with the added concentrated pseudovirus were placed at 4°C for 20 min, and real-time fluorescence imaging of live cells was performed using a double-disc laser confocal microscope (Dragonfly 200).

Image processing and analysis Images were processed in LAS X (Leica) and Imaris 9.8 (Oxford Instruments) software. Mander’s colocalization coefficient was used to describe the percentage of STX6 fluorescent signal or S-EGFP pseudovirus colocalized with different endocytic markers by setting proper thresholds for both channels to avoid background signal.

### Statistical analyses

The data are presented as the mean ± SD using Prism Version 6 (GraphPad). Statistical significance between groups was determined using Student’s *t*-test or an analysis of variance as indicated with 95% confidence intervals. The results of qRT-PCR were converted into β-actin expression to allow the relative gene expression of each sample to be presented within the same set of data.

## Data Availability

All data relevant to the study are included in the article or uploaded as supplemental material.

## SUPPLEMENTAL MATERIAL

Supplemental material jvi.00002-25-s0001.docx Tables S1 to S3 and Figures S1 to S4.

Video S1 jvi.00002-25-s0002.avi Fusion process of mCherry-STX6-labeled vesicle with S-EGFP pseudovirus vesicle.

ASM does not own the copyrights to Supplemental Material that may be linked to, or accessed through, an article. The authors have granted ASM a non-exclusive, world-wide license to publish the Supplemental Material files. Please contact the corresponding author directly for reuse.
