# Supplementary material for: Syntaxin-6 restricts SARS-CoV-2 infection by facilitating virus trafficking to autophagosomes
Source: J Virol. 2025 Apr 25;99(5):e00002-25. doi: 10.1128/jvi.00002-25 (PMC12090716; doi:10.1128/jvi.00002-25)
Supplement: Supplemental material — Tables S1 to S3 and Figures S1 to S4. [file jvi.00002-25-s0001.docx]

**Supplementary data**

Table S1-S3 and Figure S1-S4

Video S1. The fusion process of mCherry-STX6-beared vesicle with S-EGFP pseudovirus vesicle

**Supplementary Tables**

**Table S1. Main Primers used to Cloning**

| Expression plasmid | Orientation | Sequence (5’-3’) |
| --- | --- | --- |
| ACE2-TurboID | F | ATGTCAAGCTCTTCCTGGCTCC |
|  | R | CTACTGCAGCTTTTCGGCAGACCGC |
| S | F | ATGTTTGTTTTTCTTGTTTTATTGCC |
|  | R | TTATGTGTAATGTAATTTGACTCCTTTGAGC |
| Flag-STX6 | F | CCGCTCGAGATGTCCATGGAGGACCCCTTC |
|  | R | CGGGGTACCTCACAGCACTAAGAAGAGGATGAGC |
| LAMP1-Flag | F | CCGGAATTCatggcggcccccggcagc |
|  | R | CCGCTCGAGgatagtctggtagcctgcgtgac |
| TMPRSS2-Flag | F | CCGGAATTCgaacctgggcgcctgggac |
|  | R | CCGCTCGAGgccgtctgccctcatttgtcg |

**Table S2. The siRNA sequences used in RNA interference (RNAi)**

| Gene | Species | Sequence (5’-3’) |
| --- | --- | --- |
| ACE2 | Human | CUGGGAUGCACAGAGAAUATT |
| AXL | Human | GAUGGACUGUAUGCCUUGATT |
| STX6^a^ | Human | CACCAACGAGCTGAGAAATAA |

^a^Other siRNAs were brought from RIBOBIO, Guangzhou.

**Table S3. qPCR/RT-PCR primers for gene expression detection and quantification**

| Gene | Species | Sequence (F/R 5’-3’) |
| --- | --- | --- |
| ACE2 | Human | ACAGTCCACACTTGCCCAAAT/TGAGAGCACTGAAGACCCATT |
| AXL | Human | ATCTACAATGGGGACTACTACCG/GAAGGACCACACATCGCTCT |
| STX6 | Human | ACAGCAACAAGGGAAGAAATCG/ TTTCAGCTAATGCCTGCACAGA |
| β-actin | Human | CATCCTGCGTCTGGACCT/ TCAGGAGGAGCAATGATCTTG |
| N | SARS-CoV2 | GGGGAACTTCTCCTGCTAGAAT/ CAGACATTTTGCTCTCAAGCTG |
| ORF1ab | SARS-CoV2 | CCCTGTGGGTTTTACACTTAA/ ACGATTGTGCATCAGCTGA |
| G | VSV | GTGGGATGACTGGGCTCCAT/ CTGCGAAGCAGCGTCTTGAA |
| 5’UTR | HCV | Atcactcccctgtgaggaact/ gcgggttgatccaagaaagg |
| 5’UTR | EV71 | CCCTGAATGCGGCTAATCC/ATTGTCACCATAAGCAGCCA |
| N | 229E | GGCAAACGGGTGGATTTGTC/ CGCCTAACACCGTAACCTGT |
| N | OC43 | AGCAACCAGGCTGATGTCAATACC/AGCAGACCTTCCTGAGCCTTCAAT |
| **The follow sequences are used for ssqRT-PCR:** | | |
| RT (+)-N-P | | GACCTGGATAGGCTGTGTGATA*CAGACATTTTGCTCTCAAGCTG |
| qPCR (+)-N-R | | GACCTGGATAGGCTGTGTGATA* |
| qPCR (+)-N-F | | GGGGAACTTCTCCTGCTAGAAT |
| RT (-)-N-P | | ACAGCACCCTAGCTTGGTAG*GGGGAACTTCTCCTGCTAGAAT |
| qPCR (-)-N-F | | ACAGCACCCTAGCTTGGTAG* |
| qPCR (-)-N-R | | CAGACATTTTGCTCTCAAGCTG |
| *Tag sequence, non-viral genome sequences | | |

**Supplementary Figures**


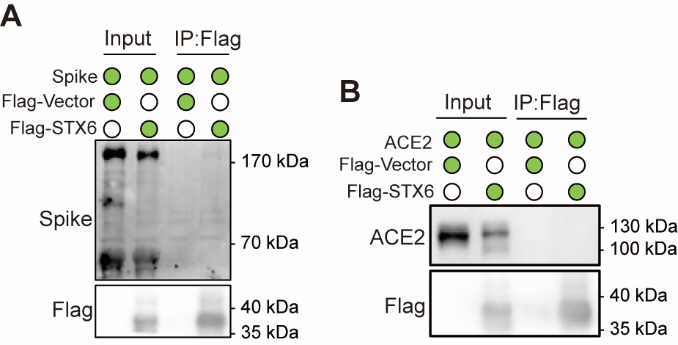


**Figure S1.** Co-immunoprecipitation analysis of syntaxin-6. (A-B). HEK-293T cells were transfected with vector, Flag-STX6 and Spike (A) or ACE2 (B). 48 hours later, cells were lysed and co-immunoprecipitation were performed with anti-Flag antibody.


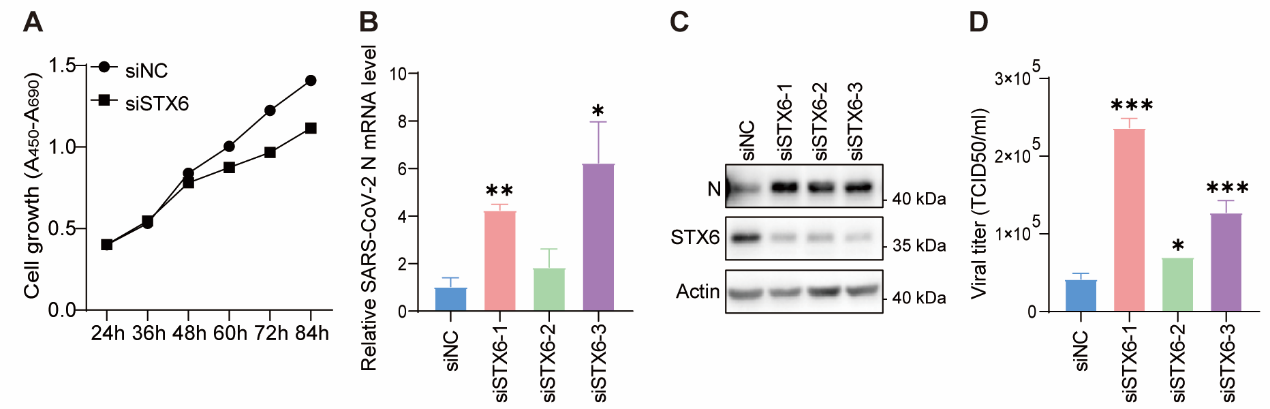


**Figure S2.** Syntaxin-6 inhibit SARS-CoV-2 infection. (A) H1299-ACE2 cells were transfected with indicated siRNAs, the cell growth was monitored by WST-1 every 12 h. (B-D) H1299-ACE2 cells were transfected with siRNAs, then infected with SARS-CoV-2 at MOI of 0.1. The virus N mRNA level, STX6 and N protein level in cells and the virus titer in supernatant were quantified at 24 hpi. The differences between two groups were determined by two-tailed t tests. Data are shown as the mean ± s.d. *p< 0.05, **p< 0.01, ***p< 0.001; ns, not significant; n=3.


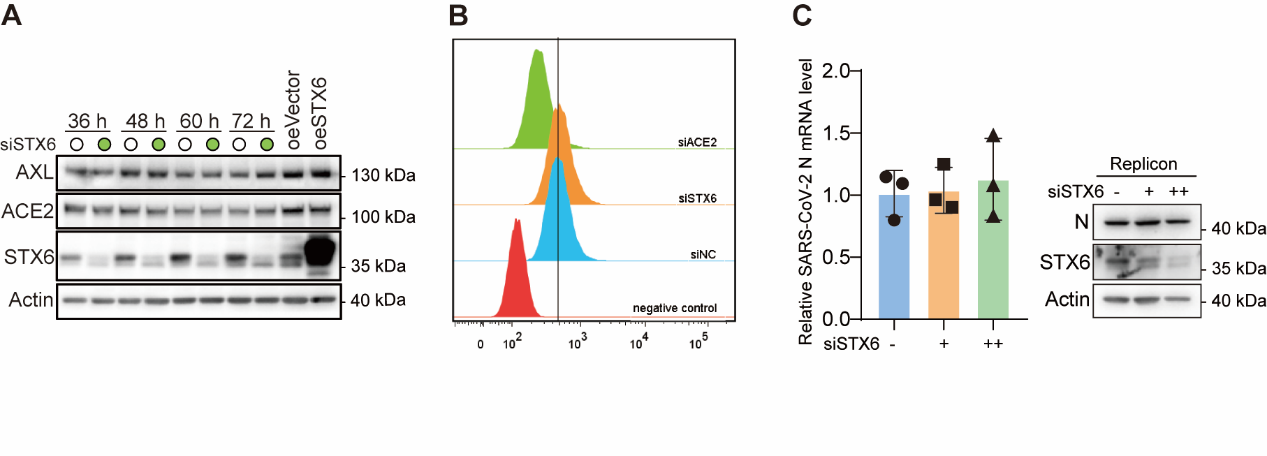


**Figure S3.** Syntaxin-6 had no effect on the expression of ACE2 and AXL. (A) H1299-ACE2 cells were transfected with indicated siRNAs or plasmids. The expression level of AXL, ACE2 and STX6 were detected by western blotting. (B) The surface distribution of ACE2 in different siRNA transfected H1299-ACE2 cells were analyzed by flow cytometry analysis. (C) H1299-ACE2 cells were transfected with siRNAs for 36 h, then transfected with SARS-CoV-2 replicon RNA. After 24 hours, the mRNA level and protein expression were measured by qRT-PCR (right) and Western blotting (left) respectively. The differences between two groups were determined by two-tailed t tests. Data are shown as the mean ± s.d. *p< 0.05, **p< 0.01, ***p< 0.001; ns, not significant; n=3.


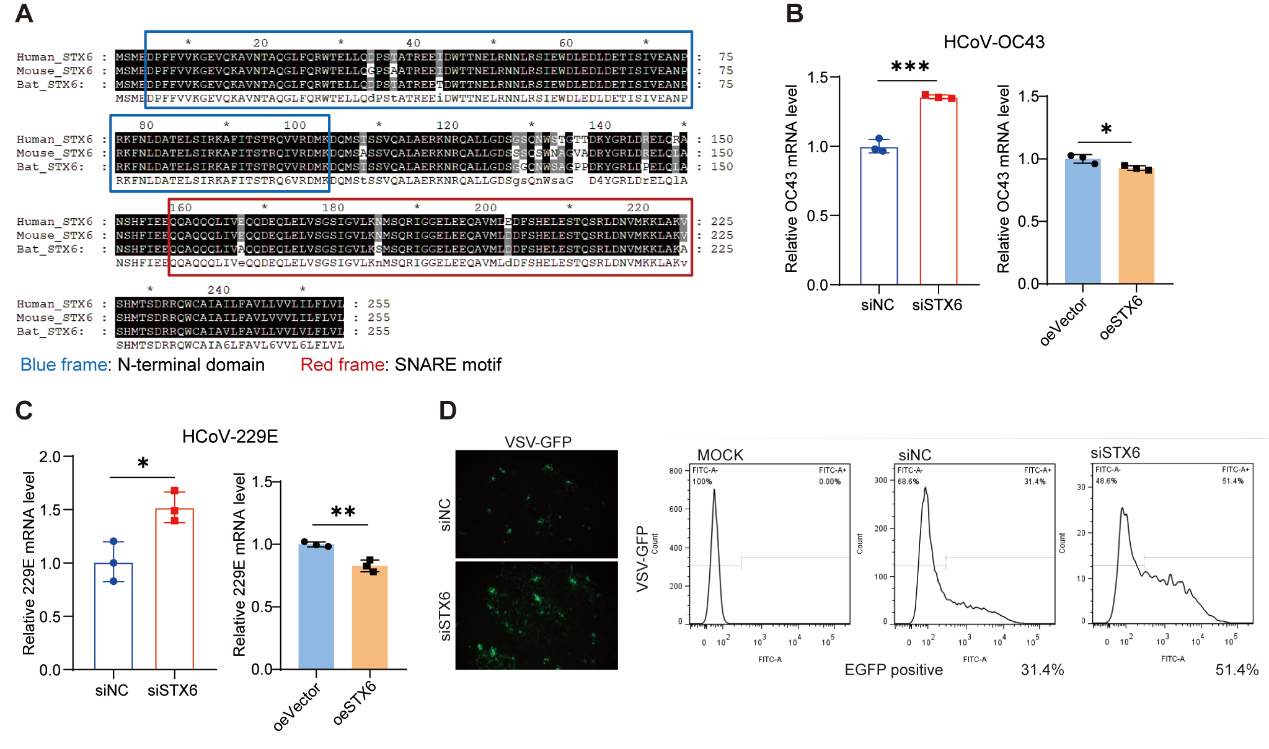


**Figure S4.** Syntaxin-6 has an antiviral activity against endocytic intrusion viruses. (A) Multiple sequence alignment of human (*Homo sapiens*), mouse (*Mus musculus*) or bat (*Rhinolophus sinicus*) orthologues of STX6. Blue frame, N-terminal domain. Red frame, SNARE motif. (B-C) H1299-ACE2 cells were transfected with control siNC, siSTX6 for 36 h (left in B and C), or transfected with vector, flag-STX6 plasmid for 24 h (right in B and C) and then infected with HCoV-OC43 (B) or HCoV-229E (C) at MOI of 0.01. The virus N mRNA level of HCoV-OC43 or HCoV-229E was quantified at 24 hpi. (D) The fluorescence intensity of GFP was observed under a microscope (magnification times, 10 × 10) and analyzed by flow cytometry after 8h post VSV-GFP infection. The differences between two groups were determined by two-tailed t tests. Data are shown as the mean ± s.d. *p< 0.05, **p< 0.01, ***p< 0.001; ns, not significant; n=3.
